# Study on Failures to Disclose Conflicts of Interest in *Environmental Health Perspectives*

**DOI:** 10.1289/ehp.112-a794b

**Published:** 2004-10

**Authors:** Merrill Goozner

**Affiliations:** Integrity in Science Project, Center for Science in the Public Interest, Washington, DC, E-mail: mgoozner@cspinet.org

The Center for Science in the Public Interest (CSPI) recently investigated conflict of interest disclosures in a cross-section of leading scientific and medical journals, including *EHP,* to determine adherence to their own policies.

*EHP*’s conflict of interest disclosure policy (EHP 2003) outlines a comprehensive list of “competing financial interests” that an author must disclose along with a published article. They include “grant support, employment (recent, present, or anticipated), … travel, consultancies, advisory board positions, patent and royalty arrangements, stock shares, … and the like.” It limits disclosure to situations where an author “may gain or lose financially through publication.” The editors also eschew any effort at enforcement, relying instead on the veracity of authors. *EHP* encourages its readers to scrutinize disclosure statements and offers to publish letters that address alleged inaccuracies.

During the study period of December 2003 through February 2004, *EHP* published 37 scientific studies. Only 2 of the studies indicated they were funded by industry, and only these 2 studies included conflict of interest disclosure statements for at least some of the authors.

The CSPI investigated the first and last authors involved in the 35 studies who did not disclose conflicts of interest. Our investigation revealed at least 3 articles (8.6%) where either the first or last authors should have disclosed conflicts in accordance with the disclosure policy.

First, a Procter and Gamble (P&G) scientist, William Owens, was identified only as a representative of the Organisation for Economic Co-operation and Development. The article (Yamasaki et al. 2003) validated an assay that may be used on P&G products. Owens did not disclose his corporate affiliation in this article, despite having disclosed his P&G employment in a previous *EHP* article (Owens and Köeter 2003).

Second, a Quebec, Canada, group led by Pierre Ayotte of CHUQ-Laval University Medical Center studied the effects of organochlorines and methyl mercury on a remote coastal population (Bilrha et al. 2003). Although there was no disclosure of a conflict of interest, the study was funded in part by the Canadian Network of Toxicology Centers, which is funded in part by the Canadian Chemical Producer Association, an industry trade group. Several of Ayotte’s previous studies were funded in part by the Canadian Chemical Producer Association and the Canadian Chlorine Coordinating Committee, although Ayotte was not directly compensated for this work.

The third group of authors who did not disclose conflicts of interest are scientists at Macquarie University who investigated the sources of lead in children near a zinc–lead smelter (Gulson et al. 2004). Brian Gulson, a professor in Macquarie’s graduate school of the environment, did not disclose that he is listed as an adviser on the website of a consulting group that advised Pasminco Ltd. (Laboratory Quality Management Services Pty Ltd. 2003), the company that ran the smelter. In a subsequent e-mail communication, Gulson informed the CSPI that one of his coauthors, Karen Mizon, was the wife of the owner of the consulting group.

In each of those cases, at least one of the authors of a study may gain financially from publication of the article, thus meeting the test of the *EHP* policy. Owens was directly employed by a company that might be affected by his findings. Gulson’s colleagues stood to gain financially from a company directly affected by the subject matter of their articles.

Ayotte’s study, although a borderline case, should have contained a disclosure statement because he had collaborated on industry-funded studies in the past and the study in question was funded by an industry-supported group. When a field of research is so closely tied to an industry, future funding for research may involve the goodwill of that industry. The spirit of conflict of interest disclosure is best served by full disclosure in such cases.

A fourth case of nondisclosure was not included in our results. In February 2004, *EHP* published a study funded by the National Institutes of Health that claimed polychlorinated biphenyls (PCBs) inhibit a receptor involved in clearing foreign substances from humans (Tabb et al. 2004). One of the authors, Bruce Blumberg, of the Department of Developmental and Cell Biology at the University of California, Irvine, is listed as a co-inventor on a patent granted in 2002 on the gene for an unrelated receptor in frogs. The use claimed for the frog gene is that it may be useful in identifying compounds that affect the receptor.

Should Blumberg disclose that patent as a potential conflict of interest? In an e-mail communication, he defended his failure to disclose by asking, “Do you seriously believe that a patent for a *Xenopus* (frog) nuclear receptor has any bearing on a paper about a mammalian receptor with an entirely different function?” Strict conflict of interest policies would argue “yes,” because it is impossible to predict how the information in the new study will affect the use of previous knowledge or what inventions considered irrelevant today may be extremely useful (and lucrative) in the future. Even though *EHP*’s policy (EHP 2003) says “may gain,” we did not count his nondisclosure in our statistics.

Judging from the findings of our limited survey, a significant percentage of articles published in *EHP* (8.6%) fail to disclose relevant conflicts of interest of authors. We cannot determine whether authors are not disclosing the relevant information to *EHP,* or if they are providing the information but *EHP* is not publishing the disclosures. Considering the importance of providing readers with such information, it would seem that *EHP* needs to develop mechanisms to minimize failures to provide “full disclosure of competing financial interests” (EHP 2003). One possible mechanism for improving compliance would be for *EHP* not to accept for 3 years any papers submitted by authors who failed to disclose information about conflicts of interest.

I look forward to your response.
